# Metformin Partially Attenuates Simvastatin‐Induced Myotoxic Responses in C2C12 Myotubes Through Metabolic Adaptation

**DOI:** 10.1096/fj.202600077RRR

**Published:** 2026-06-26

**Authors:** Chuqi He, Mike Wesselink, Jelle Y. Huijts, Zhenjia Zhong, Moritz Eggelbusch, Richard T. Jaspers, Rob C. I. Wüst

**Affiliations:** ^1^ Department of Human Movement Sciences, Faculty of Behavioural and Movement Sciences, Amsterdam Movement Sciences Vrije Universiteit Amsterdam Amsterdam the Netherlands; ^2^ Professorship of Exercise Biology, Department of Health and Sport Sciences, TUM School of Medicine and Health Technical University of Munich Munich Germany

**Keywords:** AMPK‐driven metabolic remodeling, metformin, mitochondrial dynamics, muscle atrophy, myotoxicity, statin‐associated muscle symptoms

## Abstract

Metformin is the first‐line therapy for type 2 diabetes mellitus and is commonly co‐administered with statins for cardiovascular risk reduction. However, statins can cause statin‐associated muscle symptoms, while metformin itself exerts complex effects on skeletal muscle. Because both drugs influence cellular energy metabolism and stress‐response pathways in skeletal muscle, their combined effects on muscle cells warrant investigation. C2C12 myotubes were treated with metformin (50 or 1000 μM) in the absence or presence of simvastatin (10 μM) for 24 h. Myotube morphology, differentiation, and fusion indices, myoblast proliferation, and expression of atrophy‐, stress‐, and metabolism‐related genes were assessed. Phosphorylation of key metabolic and anabolic signaling proteins (AMPK/ACC and Akt/mTOR–p70S6K) was analyzed. Mitochondrial respiration was measured using Seahorse respirometry, and mitochondrial network organization was quantified by live‐cell imaging. Simvastatin significantly reduced myotube diameter (*p* < 0.0001), impaired myogenic progression in differentiated myotubes (differentiation index, *p* < 0.0001; fusion index, *p* = 0.0152), and inhibited myoblast proliferation (*p* = 0.003). Simvastatin increased the atrophy markers (*Trim63, Fbxo32*), stress marker (*Perk*), and concurrently suppressed myogenic (*Myod*) and anabolic (p‐p70s6k/p70s6k) activity. Simvastatin also induced a broad suppression of mitochondrial and glycolytic metabolism, accompanied by reduced expression of the metabolic genes (*Glut4, Hk2*) and disruption of mitochondrial network connectivity. Co‐exposure with metformin significantly attenuated simvastatin‐induced effects, increasing myotube diameter (1.43‐fold at low dose, *p* = 0.0223, and 1.48‐fold at high dose, *p* = 0.0131), differentiation index (low dose: 1.63‐fold; high dose: 1.80‐fold; both *p* < 0.0001), and fusion index (low dose: 1.35; high dose: 1.50‐fold; both *p* < 0.01). Compared with simvastatin alone, co‐treatment with high‐dose metformin increased AMPK and ACC phosphorylation and further suppressed mTOR signaling without amplifying atrophy‐related gene expression. Despite deeper suppression of metabolic parameters (routine respiration, ATP production, *Hk2* expression), metformin preserved mitochondrial network structure, increased Ppargc1a expression, and reduced cellular stress markers (*Hri, Perk, Atf4*). Simvastatin induced metabolic suppression, mitochondrial dysfunction, and atrophy‐related responses in skeletal muscle cells. Metformin partially attenuated these alterations by preserving myotube structural integrity and reducing cellular stress signaling despite further metabolic suppression. These findings suggest that metformin may promote adaptive metabolic responses that enhance cellular resilience during simvastatin‐induced metabolic stress.

## Graphical abstract

Simvastatin induces metabolic suppression, mitochondrial dysfunction, and atrophy‐related responses in skeletal muscle cells. In differentiated C2C12 myotubes, metformin partially attenuates these alterations by preserving myotube structural integrity, reducing cellular stress signaling, and maintaining mitochondrial network organization despite further metabolic suppression. These findings suggest that metformin may promote adaptive metabolic responses that enhance cellular resilience during simvastatin‐induced metabolic stress.

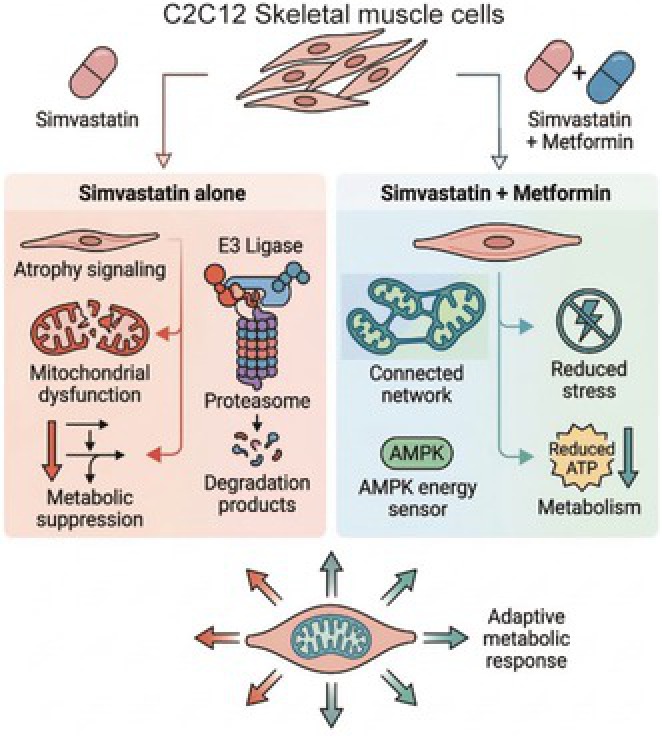

## 1 Introduction

Polypharmacy, defined as the use of five or more medications, is common among elderly individuals who take multiple medications per day for disease treatment and prevention [1, 2]. However, accumulating evidence suggests that polypharmacy may contribute to functional decline and increased vulnerability in aging populations, including adverse effects on skeletal muscle health [3, 4, 5]. The interplay between chronic disease, polypharmacy, and inactivity can create a vicious cycle impairing skeletal muscle health that requires clinical attention [6, 7]. Therefore, it is crucial to gain a molecular understanding of how commonly prescribed drugs interact when used in combination and whether such interactions exacerbate or mitigate adverse effects on skeletal muscle, particularly as populations age and preventive medications become more prevalent. A common pharmacological combination is the co‐administration of statins and metformin. Because type 2 diabetes mellitus is frequently accompanied by dyslipidemia, these drugs are commonly prescribed together to manage both glycemic control and lipid levels [8].

Statins, the most widely prescribed therapy for hypercholesterolemia, lower LDL‐cholesterol and reduce cardiovascular disease risk primarily through inhibition of 3‐hydroxy‐3‐methylglutaryl coenzyme A (HMG‐CoA) reductase [9]. Despite their benefits, up to 20%–30% of patients develop statin‐associated muscle symptoms, including myopathy, weakness, and rarely rhabdomyolysis [10, 11, 12]. Statin‐associated muscle symptoms often lead to discontinuation, paradoxically increasing cardiovascular risk [13]. Proposed mechanisms include impaired mitochondrial function, disrupted PI3K/Akt signaling, and upregulation of ubiquitin ligases such as Trim63 (MuRF1) and Fbxo32 (MAFbx/Atrogin‐1) [14].

Metformin, the most widely prescribed therapy for type 2 diabetes mellitus, lowers hepatic gluconeogenesis and enhances peripheral glucose uptake, an effect often associated with activation of AMP‐activated protein kinase (AMPK) [15]. AMPK functions as a key cellular energy sensor that coordinates metabolic responses to energetic stress [16]. However, the effects of metformin on skeletal muscle are complex and context dependent. While metformin has been reported to improve mitochondrial metabolism and metabolic health in some settings, it has also been shown to blunt resistance training‐induced hypertrophy in older adults [17] and suppress muscle growth in animal models [18, 19]. These apparently opposing effects likely reflect the dual role of AMPK‐related signaling, which can promote mitochondrial maintenance and metabolic regulation while simultaneously inhibiting mTOR‐mediated protein synthesis [20]. In addition, metformin has been reported to increase expression of atrophy‐related genes such as Trim63 and Fbxo32 under certain conditions [21].

These dual effects indicate that metformin influences multiple metabolic and stress‐related signaling pathways in skeletal muscle through AMPK‐associated signaling [22, 23, 24]. Importantly, several of these pathways overlap with mechanisms implicated in statin‐induced muscle toxicity, particularly those related to mitochondrial function, cellular energy balance, and metabolic stress responses [25]. Because of these partially overlapping pathways, metabolic adaptations induced by metformin may modify cellular responses to statins. A recent study reported that concurrent statin use paradoxically attenuated the blunted hypertrophic response to resistance training seen with metformin alone [26]. This highlights the potential for complex drug–drug interactions on skeletal muscle. However, the impact of metformin on statin‐induced muscle alterations remains poorly understood.

Here, we studied how metformin influences simvastatin‐induced alterations in skeletal muscle cell structure and function. Cell proliferation was assessed in C2C12 myoblasts, whereas myotube morphology, gene expression, protein signaling, and mitochondrial function were examined in differentiated C2C12 myotubes. This integrated approach allowed us to evaluate whether metformin modifies structural, metabolic, and stress‐related responses induced by simvastatin in skeletal muscle cells.

## 2 Methods

### 2.1 Cell Culture

C2C12 mouse myoblast cells (ATCC CRL‐1772, American Type Culture Collection, Manassas, VA, USA) were cultured in growth medium consisting of Dulbecco's Modified Eagle's Medium (DMEM, 4.5 g/L glucose) (Gibco, 11995, Thermo Fisher Scientific, Waltham, MA, USA), supplemented with 10% fetal bovine serum (FBS, Biowest, S181B, Nuaillé, France), 100 μg/mL carbenicillin/ampicillin (C1389, A0166, Sigma‐Aldrich, St. Louis, MO, USA), and 0.5% fungizone (A2942, Sigma‐Aldrich, St. Louis, MO, USA). We avoided the use of streptomycin since it affects mitochondrial function [27, 28]. Cells were grown in 75 cm^2^ culture flasks (Sigma‐Aldrich, St. Louis, MO, USA) and were passaged upon reaching approximately 50% confluency. Cells were washed twice with Dulbecco's Phosphate‐buffered saline (PBS) and passaged using 0.05% trypsin–EDTA (25200056, Thermo Fisher Scientific, Waltham, MA, USA) in PBS, followed by neutralization with complete growth medium containing 10% FBS. Cells were maintained in a humidified atmosphere containing 5% CO_2_ and 95% air at 37°C. Myotubes were generated using myoblasts with a maximal passage number of < 15.

Prior to myotube differentiation, 5 × 10^4^ myoblasts were seeded on a 12‐well plate to reach a confluency of 70%–80% after 2 days. Myoblasts were washed with PBS, and the medium was switched to differentiation medium (DMEM supplemented with 2% horse serum (HyClone, 10407223, Cytiva, Marlborough, MA, USA), 100 μg/mL carbenicillin/ampicillin, and 0.5% fungizone), and myoblasts were differentiated into myotubes for 4 days, as confirmed by microscopy.

### 2.2 Myotube Treatments

**FIGURE 1:**
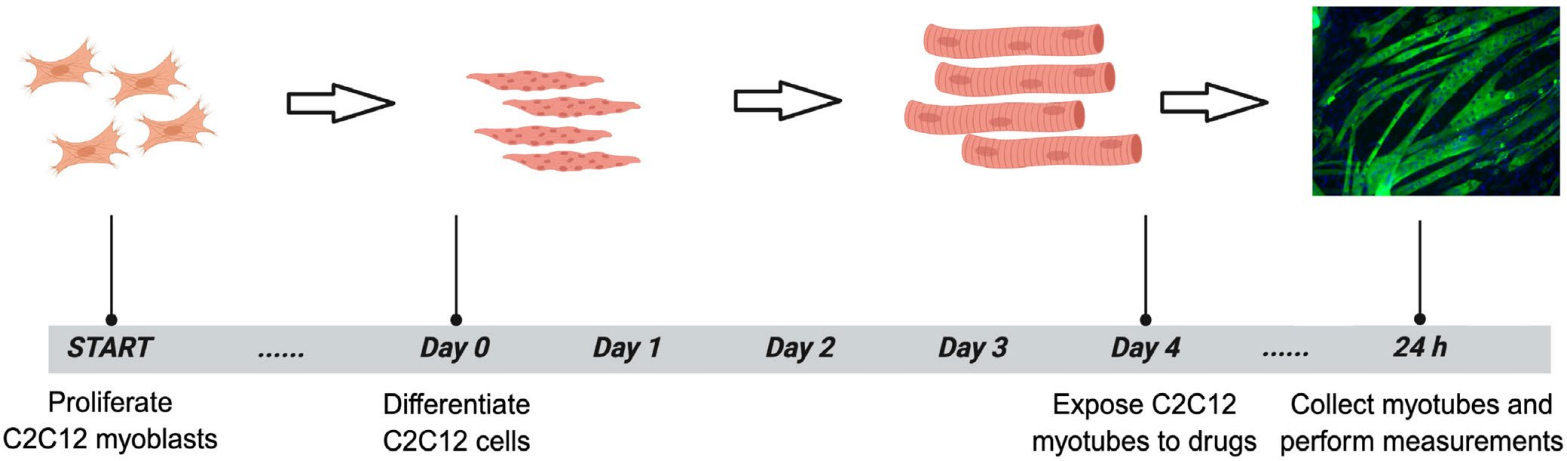
Timeline of the myotube treatment. C2C12 myoblasts (mouse skeletal muscle progenitor cells) were cultured and differentiated into myotubes. Myotubes were treated with simvastatin (10 μM, Sim) and/or metformin (50 μM, Met50 or 1 000 μM, Met1000) for 24 h.

At day 4 of differentiation, simvastatin and/or metformin were added to the culture medium for 24 h without medium change during the exposure period. (see Figure 1 for experimental overview). A single concentration of simvastatin was used (10 μM, Sim), and two different concentrations of metformin were used (50 μM, Met50 or 1000 μM, Met1000). Exposure to simvastatin was combined with either 1 000 μM or 50 μM metformin. The selected concentrations and exposure durations were guided by previous reports: Simvastatin at 10 μM has been shown to reliably induce myotoxicity in C2C12 cells [29]. Metformin at 50 μM (Met50) represents a clinically relevant concentration, approximating the upper range of plasma levels in treated patients, to test for protection under therapeutically achievable conditions. Metformin at 1 000 μM (Met1000) is a supraphysiological, mechanistic dose commonly used in vitro to potently activate pathways like AMPK, thereby clarifying the involved signaling pathways [30, 31]. Moreover, effects are robustly detectable within 24 h of simvastatin or metformin exposure [29, 32].

### 2.3 Myotube Diameter

To investigate whether metformin protects against the cytotoxic effects of simvastatin, myotube diameter was measured. Myoblasts were cultured on a 12‐well plate, differentiated for 4 days, and exposed to the compounds for 24 h. After compound exposure, myotubes were visualized at 10 × 0.3 magnification using the AxioCam MRc5 (Carl Zeiss Microscopy, Jena, Germany), and three images per well were taken. Myotube diameters were measured in at least 50 myotubes per biological replicate at 3 equidistant locations along the length of the cell. Analyses were performed using Fiji (ImageJ, National Institutes of Health, Bethesda, MD, USA).

### 2.4 Immunofluorescence

To determine myotube differentiation characteristics and to confirm myotube diameter measurements, myotubes were stained for myosin heavy chain (MHC). Myoblasts were cultured on a 12‐well plate, differentiated for 4 days, and exposed to the compounds for 24 h. After compound exposure, the differentiation medium was removed, and cells were washed with Dulbecco's PBS, fixed using 4% paraformaldehyde, washed, and permeabilized with 0.5% Triton X‐100 (Sigma‐Aldrich, St. Louis, MO, USA) in Dulbecco's PBS at room temperature (RT) for 5 min. Blocking was performed with 5% normal goat serum for 1 h at RT, and cells were stained with a mouse monoclonal anti‐MHC antibody (MF‐20, d1:50, Developmental Studies Hybridoma Bank, Iowa City, IA, USA) overnight at 4°C. Hereafter, cells were incubated with a goat anti‐mouse secondary antibody (A21141, Thermo Fisher Scientific, Waltham, MA, USA), and nuclei were visualized with DAPI (VECTASHIELD, H‐1200, Vector Laboratories, Burlingame, CA, USA). Four images per well were acquired using a fluorescence microscope at 10× magnification (ZEISS Axiovert 200 M, Carl Zeiss Microscopy, Jena, Germany). Myosin‐positive myotubes were visualized in channel 488 (green), and nuclei were visualized with DAPI in channel 405. At least 10 myotubes were measured per well using Fiji (ImageJ, National Institutes of Health, Bethesda, MD, USA). Total nuclei number, number of nuclei within myotubes, and myotube number were counted to calculate the differentiation index (differentiated nuclei/total nuclei) and fusion index (nuclei within fused myotubes/total nuclei).

### 2.5 Myoblast Proliferation

To investigate whether the medications affected myoblast proliferation, EdU staining was performed on myoblasts using the Click‐iT EdU Imaging Kit (C10640, Thermo Fisher Scientific, Waltham, MA, USA). Approximately 12000 cells per well were seeded in 12‐well plates. After allowing the cells to attach for 3 h, simvastatin and/or metformin were directly added to the growth medium to initiate exposure. Following 24 h of exposure, myoblasts were incubated with 10 μM 5‐ethynyl‐2′‐deoxyuridine (EdU) for 2 h. Myoblasts that tested positive for EdU were stained purple, while the cell nuclei were visualized with DAPI (VECTASHIELD, H‐1200, Vector Laboratories, USA) to visualize myonuclei, and images were taken using a fluorescence microscope at 10× magnification (ZEISS Axiovert 200 M, Carl Zeiss Microscopy, Jena, Germany). The percentage of EdU‐positive cells (with emission 350 nm/excitation 461 nm) was calculated using an average of three images per biological replicate.

### 2.6 Gene Expression by RT‐qPCR

To assess the effects of the medications on gene expression, real‐time quantitative polymerase chain reaction (RT‐qPCR) was performed. Myoblasts were cultured on a 12‐well plate, differentiated for 4 days, and exposed to the compound for 24 h. After compound exposure, myotubes were washed with ice‐cold PBS and harvested using TRIzol reagent (Thermo Fisher Scientific, Waltham, MA, USA), and total RNA was isolated using the RiboPure RNA Purification Kit (AM1924, Applied Biosystems, Foster City, CA, USA). Phase separation of the samples was performed using bromochloropropane, and RNA was isolated according to the manufacturer's protocol. To confirm if RNA isolation was successful, RNA concentration and quality were measured with the NanoDrop 2000c spectrophotometer (Thermo Fisher Scientific, Waltham, MA, USA). Total isolated RNA was converted into cDNA with the SuperScript VILO Master Mix (Applied Biosystems, Foster City, CA, USA), and each reaction mix contained 500 ng RNA. The complete reaction mix was incubated in a Thermal cycler (Applied Biosystems, Foster City, CA, USA) to convert RNA into cDNA using the following recommended manufacturer's reaction protocol: Priming for 10 min at 25°C, reverse transcription (RT) for 60 min at 42°C, RT inactivation for 5 min at 85°C, and finally held at 4°C. Following, cDNA samples were diluted 10× with RNAse‐free water. Amplification and quantification of the cDNA samples was performed using the Power up SYBR Green Master Mix on the QuantStudio 3 Real‐Time PCR System (Applied Biosystems, Foster City, CA, USA). All samples were tested in duplicate. A detailed description of forward and reverse primer sequences used to amplify target PCR products is indicated in (Table 1). A program for RT‐qPCR with 2 min at 50°C and 2 min at 95°C, 40 cycles consisting of 1 s at 95°C and 30 s at 60°C, a melting curve stage consisting of 15 s at 95°C, 1 min at 60°C, and 15 s at 95°C was used. The expression level of 18S ribosomal RNA (18S) was used as a housekeeping gene and was subtracted from the gene expression levels of target genes. 18S was used since it is known to be more consistent compared to other commonly used housekeeping genes. The comparative CT method was used, and the mean of controls was set to 1.

**TABLE 1:** Primer sequences used for q‐PCR.

Target Gene	Forward sequence	Reverse sequence
*Rn18s*	GTAACCCGTTGAACCCCATT	CCATCCAATCGGTAGTAGCG
*Trim63*	GGGCTACCTTCCTCTCAAGTGC	CGTCCAGAGCGTGTCTCACTC
*Fbxo32*	AGACTGGACTTCTCGACTGC	TCAGCTCCAACAACAGCCTTACT
*Foxo3*	GAAATGGGCAAAGCAGACCC	TGTCCACTTGCTGAGAGCAG
*Myod*	AGCACTACAGTGGCGACTCA	GCTCCACTATGCTGGACAGG
*Hk2*	TCGCATATGATCGCCTGCTT	AGAGATACTGGTCAACCTTCTGC
*Glut4*	TCCAGTATGTTGCGGATGCT	TGAAGAAGCCAAGCAGGAGG
*Pdk4*	CCTCTGAGGATTACTGACCGC	CCAAAACCAGCCAAAGGGG
*Ppargc1a*	ACACAACCGCAGTCGCAACA	GGGAACCCTTGGGGTCATTTGG
*Tfam*	GCGTGCTAAAAGCACTGGG	ACTTCGGAATACAGACAAGACTGA
*Hri*	GGGCATAGCTCGGAATTGGA	TGGTACCGAACCTCCGTCT
*Perk*	TCGCGGCAGGTCCTTG	ACGTCCAAATCCCACTGCTT
*Atf4*	CCACCATGGCGTATTAGAGG	CAACACTGCTGCTGGATTTC

### 2.7 Protein Content by Western Blot

To evaluate the protein concentration of key signaling pathways, a Western Blot was performed. Myoblasts were cultured on a 12‐well plate, differentiated for 4 days, and exposed to the compound for 24 h. After compound exposure, myotubes were lysed in ice‐cold RIPA lysis buffer containing phosphatase and protease inhibitors (Sigma‐Aldrich, St. Louis, MO, USA). The Pierce BCA Protein Assay Kit (23 225, Thermo Fisher Scientific, Waltham, MA, USA) was used to assess total protein concentration through the Epoch Microplate Spectrophotometer (BioTek Instruments, Winooski, VT, USA). Approximately 10 μg of protein was mixed with 4× Laemmli Sample Buffer (1 610 747, Bio‐Rad Laboratories, Hercules, CA, USA) supplemented with dithiothreitol (DTT; DL‐1,4‐dithiothreitol, 99%, ACROS Organics; 10 458 730, Thermo Fisher Scientific, Waltham, MA, USA). Samples were heated for 5 min at 90°C, cooled on ice, and loaded onto a 4%–20% Mini‐PROTEAN TGX Precast Gel (Bio‐Rad Laboratories, Hercules, CA, USA), except for OXPHOS samples, which were not heated prior to loading. Proteins were separated by SDS–PAGE at 50 V for approximately 15 min, followed by 150 V for 60–120 min, until the dye front reached the bottom of the gel.

A prestained protein molecular weight marker (Precision Plus Protein Dual Color Standards, 10–250 kDa, 1 610 374, Bio‐Rad, USA) was loaded in each gel to estimate protein sizes. Proteins were transferred to polyvinylidene difluoride (PVDF) membranes using a wet transfer system. PVDF membranes were first activated in 96% ethanol and equilibrated in cold transfer buffer. Transfer sandwiches were assembled with fiber pads and filter papers, and transfers were performed in transfer buffer under constant voltage (80 V) for 60 min at 4°C with cooling using ice packs.

Membranes were blocked in 2% Amersham ECL Prime Blocking Reagent (RPN418, Cytiva, Marlborough, MA, USA) prepared in TBST (TBS containing 0.1% Tween‐20) for 1 h at 4°C and incubated with primary antibodies overnight at 4°C. Primary antibodies used were anti‐p‐AMPK (Thr172) (rabbit polyclonal antibody (pAb), d1:2000, #2531, Cell Signaling Technology, USA), anti‐AMPK (rabbit pAb, d1:4000, #2532, Cell Signaling Technology, USA), anti‐p‐ACC (Ser79) (rabbit pAb, d1:1000, #3661, Cell Signaling Technology, USA), anti‐ACC (rabbit pAb, d1:1000, #3662, Cell Signaling Technology, USA), anti‐p‐AKT (Ser473) (rabbit pAb, d1:2000, #9271, Cell Signaling Technology, USA), anti‐AKT (rabbit monoclonal antibody (mAb), d1:4000, C67E7, Cell Signaling Technology, USA), anti‐p‐P70S6K (Thr389) (rabbit mAb, d1:2000, 108D2, Cell Signaling Technology, USA), anti‐P70S6K (rabbit mAb, d1:4000, #2708, Cell Signaling Technology, USA) and pan‐actin (rabbit mAb, 1:4000, D18C11, Cell Signaling Technology, USA). Membranes were washed and exposed to secondary antibody (Polyclonal Goat Anti‐Rabbit HRP, P0448, Dako, Agilent Technologies, Santa Clara, CA, USA) at room temperature for an hour. Detection of immunoreactivity signals was executed within 5 min using the Western Blotting Detection Kit ECL Select (GERPN2235, Cytiva, Marlborough, MA, USA) and the digital luminescent ImageQuant LAS 500 (GE Healthcare Bio‐Sciences, Uppsala, Sweden). Equal loading of samples was confirmed using pan‐actin as a loading control. Band intensities were quantified using ImageJ software. Total proteins were normalized to pan‐actin, and phosphorylation levels were calculated as the ratio of phosphorylated to total protein (e.g., p‐AMPK/AMPK, p‐ACC/ACC, p‐AKT/AKT, and p‐p70S6K/p70S6K).

### 2.8 Mitochondrial Respiration

To investigate the effects of exposure to simvastatin and metformin on mitochondrial respiration and glycolysis, oxygen consumption rate (OCR) and extracellular acidification rate (ECAR) were measured using a Seahorse XFe96 Analyzer (Agilent Technologies, Santa Clara, CA, USA). Myoblasts were seeded at a density of 10000 cells per well in Seahorse XF96 cell culture microplates, differentiated for 4 days, and exposed to compounds for 24 h. After compound exposure, myotubes were washed twice, and the medium was replaced with Seahorse Assay Medium (10 mM glucose, 2 mM glutamine, 1 mM pyruvate, 5 mM HEPES, and 1.3 mM bicarbonate, pH 7.4). Cells were incubated for 45 min at 37°C in a non‐CO_2_ incubator prior to the assay. Basal OCR and ECAR were recorded, followed by sequential injections of mitochondrial inhibitors according to a standard mitochondrial stress test protocol: Oligomycin (2 μM), FCCP (0.75 μM), and rotenone/antimycin A (0.5 μM each). These concentrations were selected based on our established Seahorse assay conditions for C2C12 myotubes and produced the expected responses for mitochondrial respiration, including maximal oxygen consumption following FCCP injection. Three measurement cycles were performed at baseline and after each compound injection, with each cycle consisting of mixing, waiting, and measuring phases. Non‐mitochondrial respiration measured after rotenone/antimycin A was subtracted from all OCR values. OCR and ECAR values were normalized to total protein content per well. Measurements were obtained from independent biological replicates.

### 2.9 Mitochondrial Morphology

To visualize mitochondrial networks, C2C12 myoblasts were seeded at approximately 10000 cells per well on 8‐well ibidi plates (Ibidi, Martinsried, Germany), differentiated for 4 days, and exposed to compounds for 24 h. Cells were seeded at identical densities in all wells to minimize variability in mitochondrial content between experimental conditions. After treatment, myotubes were stained with MitoTracker Green FM (M7514, Thermo Fisher Scientific, Waltham, MA, USA) for 15 min and imaged using a Nikon AXR confocal microscope (Nikon Corporation, Tokyo, Japan) with a 40× objective. MitoTracker Green fluorescence was acquired using 488 nm excitation and detected in the 500–530 nm emission range. All images were acquired using identical imaging settings across experimental conditions.

Following image acquisition, images were deconvoluted using Huygens Professional (version 22.10, Scientific Volume Imaging, Hilversum, The Netherlands). Mitochondrial morphology was quantified using the Mitochondrial Network Analysis (MiNA) macro implemented in ImageJ and executed using default automated parameters. Images were automatically thresholded and converted to binary format prior to skeletonization, generating a one‐pixel‐wide representation of the mitochondrial network. The MiNA macro was used to quantify mitochondrial footprint, mean branch length, summed branch length, and number of network branches. At least 50 cells were analyzed per biological replicate. Because MitoTracker Green labels mitochondria independently of membrane potential, the analysis focused on mitochondrial network morphology rather than fluorescence intensity‐based measurements.

### 2.10 Statistics

Data were tested for normality using the Shapiro–Wilk test and for homogeneity of variances using Levene's test. When both assumptions were met, data were analyzed using two‐way ANOVA to assess the main effects of simvastatin and metformin, as well as their interaction, followed by post hoc multiple comparison tests where appropriate. In cases where assumptions were violated, data were log‐transformed prior to analysis. Statistical significance was considered when *p* < 0.05. Statistical analyses were performed with GraphPad Prism version 10 (GraphPad Software, San Diego, CA, USA). Results are presented as mean ± standard error of the mean (SEM). Each experiment was performed using 4–8 independent biological replicates. The exact number of biological replicates (N) for each experiment is indicated in the corresponding figure legends.

## 3 Results

### 3.1 Metformin Partially Attenuates Simvastatin‐Induced Structural Impairment of Myotubes

**FIGURE 2:**
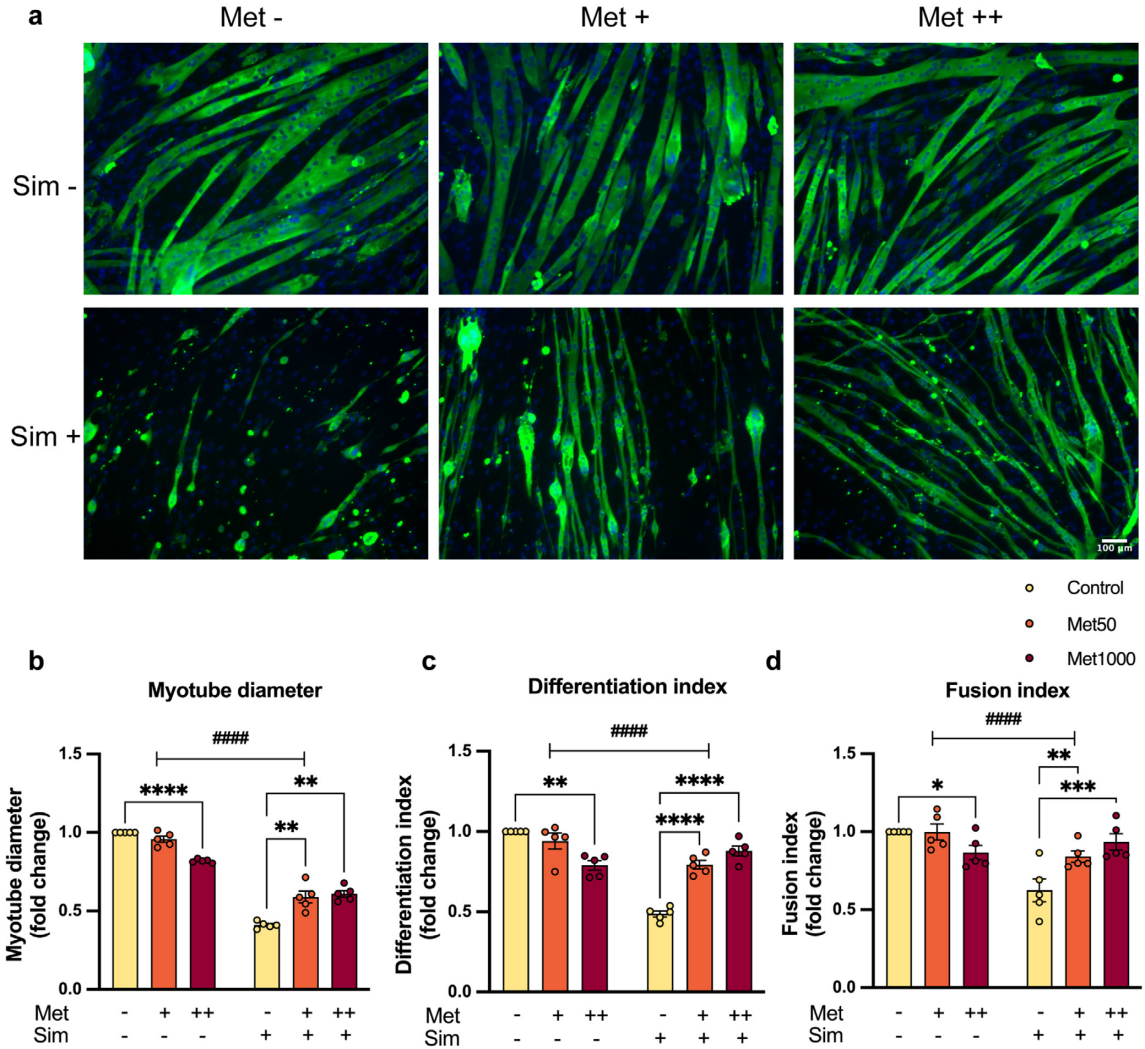
Effects of simvastatin (Sim) and metformin (Met) on C2C12 myotube diameter. (a) Representative immunofluorescence images of C2C12 myotubes stained with anti‐myosin heavy chain (MyHC, green) showing morphological changes after exposure. Scale bar: 100 μm. (b) Quantification of myotube diameter after 24‐h exposure across all experimental groups. (c) Myotube differentiation index (differentiated nuclei/total nuclei), and (d) fusion index (fused tube nuclei/total nuclei) of the myotubes after 24‐h exposure across all experimental groups. Data are presented as the mean ± SEM, *N* = 5, *p < 0.05, **p < 0.01, and ****p < 0.0001. ^#^ *p* < 0.0001 indicates a main effect of simvastatin. Significant interaction effects identified by two‐way ANOVA are described in the Results but are not indicated in the graphs.

Typical examples of myotubes cultured under the experimental conditions are shown in (Figure 2a). Control myotubes exhibited elongated, organized structures, whereas simvastatin‐exposed myotubes appeared thinner and less organized (Figure 2a). Simvastatin exposure for 24 h significantly reduced the diameter of differentiated C2C12 myotubes (main effect of simvastatin, *p* < 0.0001, Figure 2b). When combined with metformin, metformin partially rescued myotube morphology in a dose‐dependent manner (interaction effect, *p* < 0.0001). Myotube diameter was higher after both low and high doses of metformin compared with simvastatin exposure alone (1.43 to 1.48‐fold for both doses, *p* < 0.022). However, high‐dose metformin alone reduced myotube diameter compared to control (Met1000 vs. control: 0.82‐fold, *p* < 0.0001), while low‐dose metformin did not alter myotube diameter.

Simvastatin exposure also significantly reduced the differentiation index (main effect of simvastatin: *p* < 0.0001) and fusion index (main effect of simvastatin: *p* = 0.0152, Figure 2c,d). When combined with metformin, metformin enhanced both differentiation (Sim + Met50 vs. Sim, 1.63‐fold; Sim + Met1000 vs. Sim, 1.80‐fold; both *p* < 0.0001) and fusion (Sim + Met50 vs. Sim, 1.35‐fold, *p* = 0.0085; Sim + Met1000 vs. Sim, 1.50‐fold, *p* = 0.0008) relative to simvastatin alone, with significant interaction effects for both indices (interaction effect: *p* < 0.001). However, high‐dose metformin alone reduced the differentiation index and fusion index compared to control (Met1000 vs. control, 0.79‐fold, *p* = 0.0021 for differentiation index; 0.87‐fold, *p* = 0.0464 for fusion index).

### 3.2 Metformin Fails to Improve Simvastatin‐Induced Inhibition of Myoblast Proliferation

**FIGURE 3:**
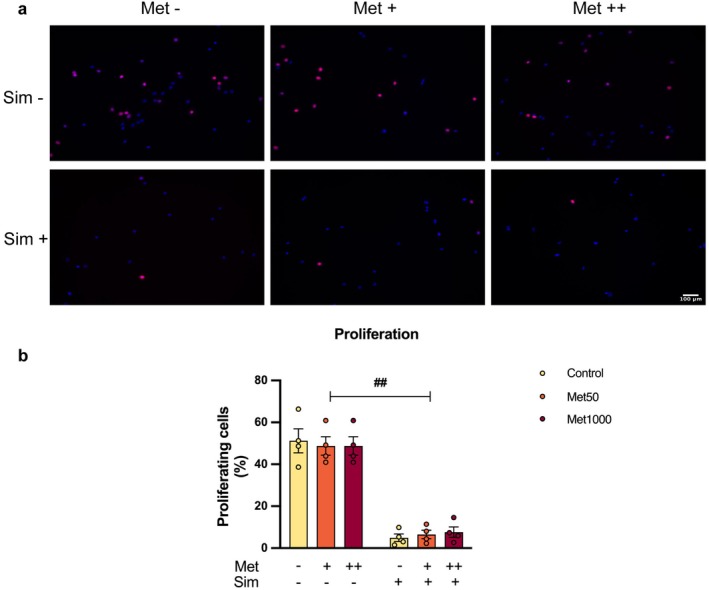
Effects of simvastatin (Sim) and metformin (Met) on C2C12 myoblast proliferation. (a) Representative images of proliferating C2C12 myoblasts after 24‐h exposure across all experimental groups. Nuclei of proliferating cells were stained in red, and nuclei of all cells were stained with Hoechst in blue. Scale bar: 50 μm. (b) Quantification of cell proliferation rate relative to control. Data are presented as the mean ± SEM, *N* = 4, **p < 0.01. ^##^ *p* < 0.01 indicates a main effect of simvastatin.

EdU staining showed that simvastatin inhibited myoblast proliferation (main effect of simvastatin, *p* = 0.003, Figure 3). Neither low‐dose nor high‐dose metformin co‐exposure restored proliferation, and metformin alone had no significant effect on proliferation.

### 3.3 Simvastatin Upregulates Atrophy‐Related Genes While Metformin Activates AMPK Signaling

**FIGURE 4:**
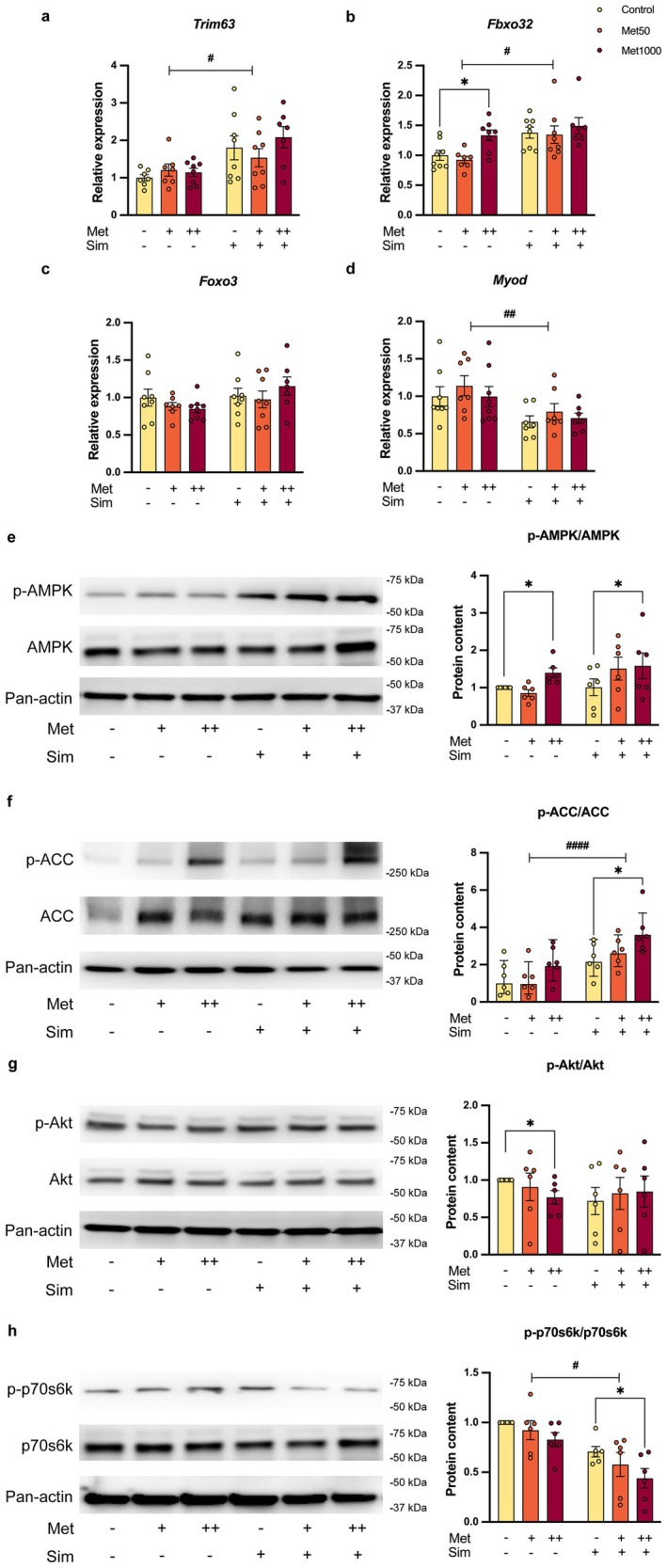
Effects of simvastatin (Sim) and metformin (Met) on muscle atrophy‐related genes and signaling protein phosphorylation in C2C12 myotubes. RT‐qPCR analysis of (a) *Trim63* (MuRF1), (b) *Fbxo32* (MAFbx/Atrogin‐1), and (c) *Foxo3*, (d) *MyoD* mRNA expression levels in C2C12 myotubes after 24‐h exposure. Western blot analysis and quantification of (e) p‐AMPK (Thr172)/AMPK ratio, (f) p‐ACC (Ser79)/ACC ratio, (g) p‐AKT (Ser473)/AKT ratio, and (h) p‐p70S6K (Thr389)/p70S6K ratio. Pan‐Actin was used as a loading control. The indicated molecular weights represent approximate band positions estimated using the Precision Plus Protein marker. Results are expressed as relative expression to control of independent experiments and represent the mean ± SEM (*N* = 6), *p < 0.05, **p < 0.01. ^a^ *p* < 0.05, ^b^ *p* < 0.01, ^c^ *p* < 0.0001 indicate a main effect of simvastatin. Significant metformin main effects and interaction effects identified by two‐way ANOVA are described in the Results but are not indicated in the graphs.

Simvastatin upregulated Tripartite motif‐containing 63 (*Trim63*, main effect of simvastatin, *p* = 0.0221, Figure 4a) and F‐box only protein 32 (*Fbxo32*, main effect of simvastatin, *p* = 0.0171, Figure 4b). High‐dose metformin reduced *Fbxo32* expression when administered alone (Met1000 vs. control, 0.67‐fold, *p* = 0.0323). However, neither dose of metformin altered *Trim63* or *Fbxo32* expression in simvastatin‐exposed myotubes. Forkhead box O3 (*Foxo3*) expression was unchanged (Figure 4c). Simvastatin also decreased Myogenic differentiation 1 expression (*Myod*, main effect of simvastatin, *p* = 0.0026, Figure 4d), which was not restored by metformin.

For AMPK‐related signaling, western blot analysis showed a significant main effect of metformin on the p‐AMPK/AMPK ratio (main effect of metformin: *p* = 0.0302), and high‐dose metformin increased the p‐AMPK/AMPK ratio both when administered alone and in combination with simvastatin (Met1000 vs. control, 1.40‐fold, *p* = 0.0274; Sim + Met1000 vs. Sim, 1.57‐fold, *p* = 0.0383; Figure 4e).

In line with this, the p‐ACC/ACC ratio, a downstream readout of AMPK signaling, was increased by metformin (main effect of metformin: *p* = 0.0109) and simvastatin (main effect of simvastatin: *p* = 0.0001), and was further increased by high‐dose metformin under simvastatin exposure (Sim + Met1000 vs. Sim, 1.61‐fold, *p* = 0.0125; Figure 4f). Corresponding p‐ACC/pan‐actin and ACC/pan‐actin data are provided in Figure S1. p‐ACC/pan‐actin was increased by metformin (main effect of metformin: *p* = 0.0315) and simvastatin (main effect of simvastatin: *p* = 0.0007), whereas ACC/pan‐actin was increased by simvastatin only (main effect of simvastatin: *p* = 0.0266), with no effect of metformin.

For Akt signaling, high‐dose metformin modestly reduced the p‐Akt/Akt ratio compared with control (Met1000 vs. control, 0.77‐fold, *p* = 0.0498), whereas neither dose affected p‐Akt/Akt in simvastatin‐exposed myotubes (Figure 4g). For p70S6K signaling, both simvastatin and metformin reduced the p‐p70S6K/p70S6K ratio (main effect of simvastatin: *p* = 0.0258; main effect of metformin: *p* = 0.0337; Figure 4h), and high‐dose metformin further suppressed p70S6K phosphorylation under simvastatin exposure (Sim + Met1000 vs. Sim, 0.62‐fold, *p* = 0.0184).

### 3.4 Metformin Further Suppresses Mitochondrial Respiration and Alters Metabolic Gene Expression Under Simvastatin Exposure

**FIGURE 5:**
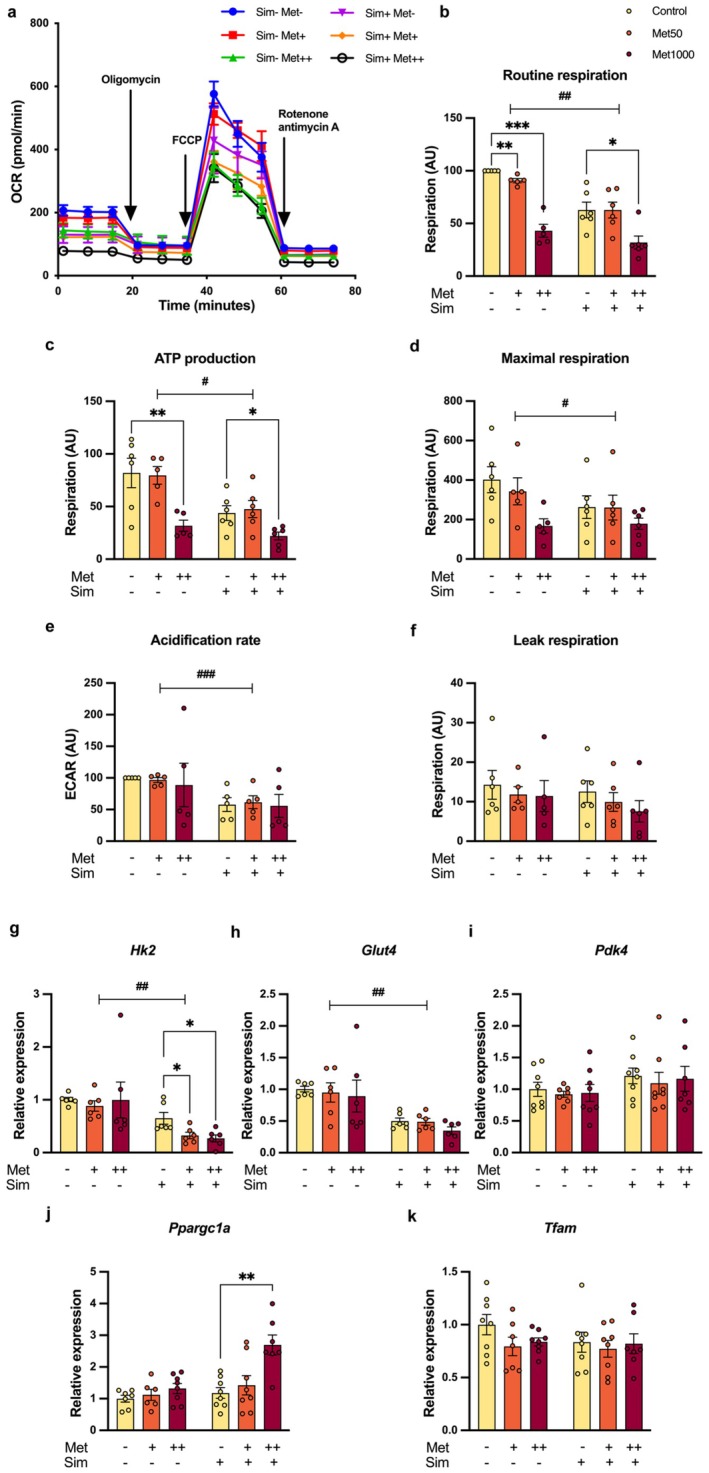
Mitochondrial respiratory function and energy metabolism gene expression following simvastatin (Sim) and metformin (Met) exposure. (a) Typical example of a mitochondrial respiration assay. (b) Baseline (routine) oxygen consumption rates (OCR). (c) The oxygen utilization rate for ATP production (routine minus leak respiration). (d) Maximal uncoupled mitochondrial respiration. (e) Extracellular acidification rates (ECAR). (f) Leak respiration. (g) *Hk2*, (h) *Glut4*, and (i) *Pdk4*, (j) *Ppargc1a*, (k) *Tfam* mRNA expression levels in C2C12 myotubes after 24‐h exposure. Results are expressed as relative expression to control of independent experiments and represent the mean ± SEM (*N* = 5–6), *p < 0.05, **p < 0.01, and ***p < 0.001. ^a^ *p* < 0.05, ^b^ *p* < 0.01, ^c^ *p* < 0.001 indicate a main effect of simvastatin. Significant metformin main effects and interaction effects identified by two‐way ANOVA are described in the Results but are not indicated in the graphs.

Oxygen consumption rate (OCR), which reflects mitochondrial oxidative metabolism, was used to assess different components of respiration (Figure 5a). Routine respiration, defined as the basal OCR under normal conditions reflecting overall mitochondrial activity (Figure 5b), was significantly lower after simvastatin (main effect of simvastatin: *p* = 0.0017) and metformin (main effect of metformin: *p* = 0.0004) exposure. High‐dose metformin reduced routine respiration (Met1000 vs. control, 0.43‐fold, *p* = 0.0007) and further suppressed it in the presence of simvastatin (Sim + Met1000 vs. Sim, 0.51‐fold, *p* = 0.0182). Oxygen consumption linked to ATP production, which is mitochondrial respiration directly associated with oxidative phosphorylation (Figure 5c), was significantly decreased by simvastatin (main effect of simvastatin: *p* = 0.0413) and metformin (main effect of metformin: *p* = 0.0014) in a dose‐dependent manner (interaction effect, *p* = 0.0275). High‐dose metformin reduced ATP (Met1000 vs. control, 0.35‐fold, *p* = 0.0053) and further suppressed ATP with simvastatin (Sim + Met1000 vs. Sim, 0.51‐fold, *p* = 0.0155). Maximal respiration (Figure 5d), which reflects the maximal electron transport chain capacity under uncoupled conditions, was significantly reduced with simvastatin (main effect of simvastatin: *p* = 0.0356), but no significant effects of metformin or interaction effects were observed. The extracellular acidification rate (ECAR), an indicator of glycolytic activity measured at baseline (corresponding to routine respiration; Figure 5e), was reduced by simvastatin (main effect of simvastatin: *p* = 0.0007). Leak respiration, representing oxygen consumption not coupled to ATP synthesis and reflecting proton leak and inner membrane integrity, was not different between conditions (Figure 5f).

Simvastatin reduced the gene expression of Hexokinase 2 (*Hk2*, main effect of simvastatin: *p* = 0.0047, Figure 5g), which was further suppressed by metformin (Sim + Met50 vs. Sim, 0.50‐fold, *p* = 0.0348; Sim + Met1000 vs. Sim, 0.41‐fold, *p* = 0.0153). Glucose transporter type 4 (*Glut4*, main effect of simvastatin: *p* = 0.0026, Figure 5h) expression was also decreased by simvastatin. For peroxisome proliferator‐activated receptor gamma coactivator 1‐alpha (*Ppargc1a*, Figure 5j), high‐dose metformin significantly increased Ppargc1a expression in simvastatin‐treated myotubes (Sim + Met1000 vs. Sim, 2.12‐fold, *p* = 0.0033), while Pyruvate dehydrogenase kinase 4 (*Pdk4*, Figure 5i) and Transcription factor A mitochondrial (*Tfam*, Figure 5k) showed no significant changes with either or both compounds.

### 3.5 Metformin Preserves Mitochondrial Network Structure Despite Metabolic Inhibition

**FIGURE 6:**
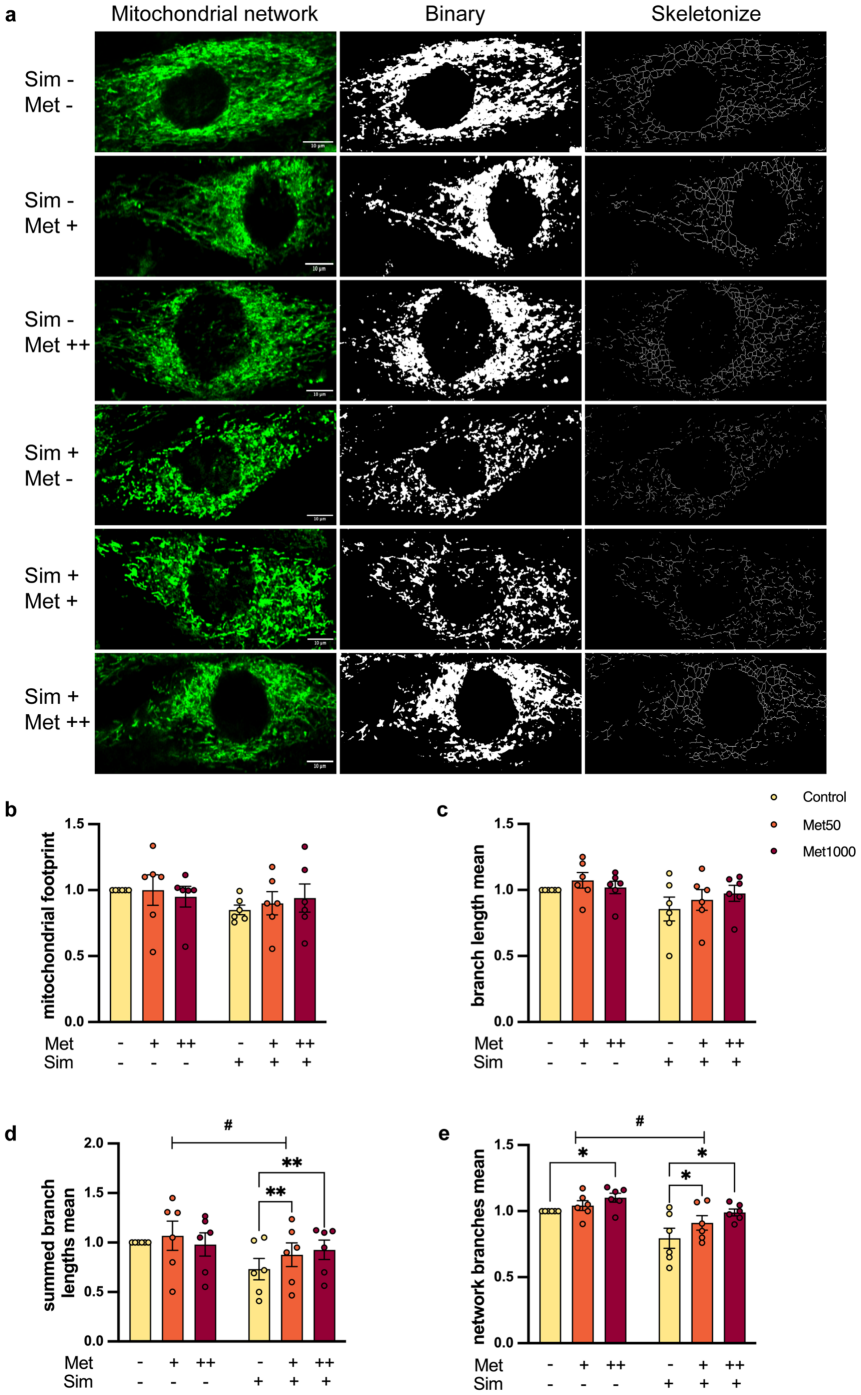
Mitochondrial network structure in C2C12 myotubes treated with simvastatin (Sim) and metformin (Met). (a) Representative fluorescence microscopy images of mitochondrial networks in C2C12 myotubes after 24‐h exposure. Mitochondria were visualized using MitoTracker staining in green. Scale bar: 10 μm. Quantitative analysis of mitochondrial network parameters: (b) Mitochondrial footprint (the area or volume of the image consumed by mitochondrial signal). (c) Branch length mean (the mean length of all the lines used to represent the mitochondrial structures). (d) Summed mean branch lengths (the mean of the sum of the lengths of branches for each independent structure). (e) Network branches mean (the mean number of attached lines used to represent each structure). Results are expressed as relative expression to control of independent experiments and represent the mean ± SEM (*N* = 6), *p < 0.05, and **p < 0.01. ^#^ *p* < 0.05 indicates a main effect of simvastatin. A significant main effect of metformin was also detected by two‐way ANOVA, but is not indicated in the graphs.

Mitochondrial network impairment refers to disruptions in the structural organization of the mitochondrial reticulum, including changes in network size, continuity, and branching characteristics. Simvastatin disrupted mitochondrial networks into shorter, disconnected fragments (Figure 6a). Metformin co‐exposure preserved network structure.

Quantitative analysis showed that neither simvastatin nor metformin affected the mitochondrial footprint or mean branch length (Figure 6b,c). Simvastatin reduced the mean summed branch length (main effect of simvastatin: *p* = 0.0180; Figure 6d), but metformin attenuated this reduction (Sim + Met50 vs. Sim, 1.20‐fold, *p* = 0.0043; Sim + Met1000 vs. Sim, 1.27‐fold, *p* = 0.0087). Simvastatin (main effect of simvastatin: *p* = 0.0411) and metformin (main effect of metformin: *p* = 0.0067) had significant effects on the mean number of network branches (Figure 6e), with opposite directions of change. Simvastatin reduced the mean number of mitochondrial network branches, whereas metformin increased it (Met1000 vs. control, 1.10‐fold, *p* = 0.0313). Both doses of metformin also increased the mean number of network branches in simvastatin‐exposed myotubes (Sim + Met50 vs. Sim, 1.15‐fold, *p* = 0.0152; Sim + Met1000 vs. Sim, 1.25‐fold, *p* = 0.0159).

### 3.6 Metformin Attenuates Simvastatin‐Induced Cellular Stress Response

**FIGURE 7:**
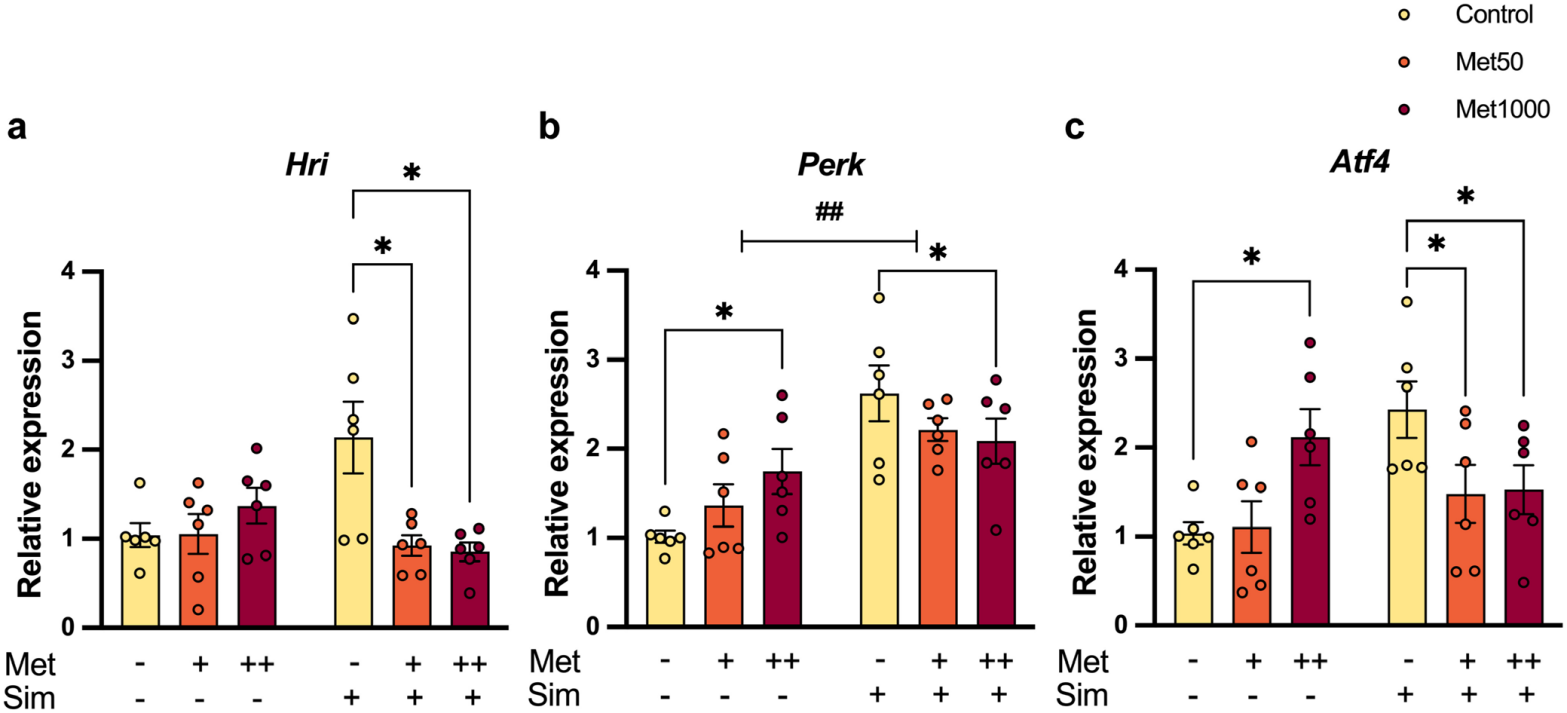
Expression of cellular stress response genes following simvastatin (Sim) and metformin (Met) exposure. RT‐qPCR analysis of stress‐related genes in C2C12 myotubes after 24‐h exposure: (a) *Hri*, (b) *Perk*, and (c) *Atf4* mRNA expression levels normalized to control. Results are expressed as relative expression to control of independent experiments and represent the mean ± SEM (*N* = 6–8), *p < 0.05. ^#^ *p* < 0.01 indicates a main effect of simvastatin. Significant interaction effects identified by two‐way ANOVA are described in the Results but are not indicated in the graphs.

We next measured gene expression levels of key cellular stress markers to test whether simvastatin and metformin would differentially alter cellular stress. Heme‐regulated eukaryotic initiation factor 2 alpha kinase (*Hri*) showed a significant interaction effect between both compounds (interaction effect: *p* = 0.0320, Figure 7a). Only in the presence of simvastatin did metformin reduce *Hri* expression at low (Sim + Met50 vs. Sim, 0.43‐fold, *p* = 0.0223) and high dose (Sim + Met1000 vs. Sim, 0.40‐fold, *p* = 0.0131).

Simvastatin increased the expression of Protein kinase R‐like endoplasmic reticulum kinase (*PERK*, main effect of simvastatin: *p* = 0.009, Figure 7b). Only the high‐dose metformin increased *Perk* expression compared to control (Met1000 vs. control: 1.73‐fold, *p* = 0.0439). However, high‐dose metformin blunted this simvastatin‐induced increase (Sim + Met1000 vs. Sim, 0.80‐fold, *p* = 0.0424).

We observed an interaction effect for the gene expression of the ER‐stress marker Activating transcription factor 4 (*Atf4*, interaction effect: *p* = 0.0052, Figure 7c). High‐dose metformin alone increased *Atf4* gene expression (Met1000 vs. control, 2.04‐fold, *p* = 0.03), but both low‐dose (Sim + Met50 vs. Sim, 0.61‐fold, *p* = 0.0404) and high‐dose (Sim + Met1000 vs. Sim, 0.63‐fold, *p* = 0.0184) metformin reduced *Atf4* gene expression in simvastatin‐exposed myotubes.

## 4 Discussion

In this study, we investigated how metformin influences simvastatin‐induced myotoxic responses in vitro. Our results show that simvastatin imposed a marked metabolic suppression associated with impaired mitochondrial function and altered mitochondrial network organization, accompanied by reduced anabolic signaling and activation of atrophy‐related pathways. These molecular alterations were associated with functional changes, including reduced myoblast proliferation, decreased differentiation and fusion capacity, and reduced myotube diameter. Metformin, particularly at higher concentrations, partially attenuated several of these simvastatin‐induced changes by preserving myotube diameter and aspects of myogenic integrity, reducing cellular stress responses, and maintaining mitochondrial network structure despite further suppression of metabolic activity. Together, these findings highlight a complex interaction between two commonly co‐prescribed medications. Simvastatin induces coordinated metabolic, stress‐related, and atrophy‐associated responses in muscle cells, whereas metformin co‐exposure modifies several of these processes. These observations suggest that metformin may promote metabolic and signaling adaptations that contribute to the structural resilience of differentiated myotubes during simvastatin exposure.

### 4.1 Simvastatin Induces Atrophy‐Related Molecular and Structural Alterations in Differentiated Myotubes

Our results indicate that simvastatin induces structural alterations in differentiated myotubes through established atrophy‐related mechanisms. Specifically, simvastatin significantly reduced myotube diameter and upregulated *Trim63* (MuRF1) and *Fbxo32* (MAFbx/Atrogin‐1) gene expression, indicating activation of the ubiquitin–proteasome pathway, a key regulator of skeletal muscle protein degradation [25, 29, 33]. These findings are consistent with those of previous reports showing that statins impair PI3K/Akt signaling and thereby promote FOXO‐mediated transcription of these E3 ligases [14, 34, 35]. The concomitant reduction in mitochondrial respiratory capacity aligns with the well‐documented statin‐induced inhibition of coenzyme Q10 synthesis, leading to mitochondrial dysfunction [36]. Furthermore, the disrupted mitochondrial network architecture observed here corroborates prior evidence of statin‐induced mitochondrial fragmentation and oxidative stress [37]. Together, these findings indicate that simvastatin promotes atrophy‐like molecular and structural changes in differentiated myotubes.

### 4.2 Metformin Partially Preserves Myotube Diameter and Structural Integrity During Simvastatin Exposure

Our finding that metformin partially attenuates the simvastatin‐induced reduction in myotube diameter is consistent with previous reports suggesting that metformin may influence muscle responses under stress conditions. Metformin has been shown to reduce IL‐1–induced atrophy in C2C12 myotubes [38] and to prevent disuse atrophy in a hindlimb suspension mouse model [39]. In addition to attenuating the reduction in myotube diameter, metformin improved both the differentiation index and fusion index during simvastatin exposure, suggesting partial preservation of myogenic characteristics.

### 4.3 AMPK‐Associated Metabolic Signaling Changes by Metformin During Simvastatin Exposure

Importantly, this structural preservation occurred despite further suppression of anabolic signaling and cellular metabolism. Simvastatin reduced p‐p70S6K phosphorylation and ATP production, and high‐dose metformin further decreased both parameters. In addition, metformin combination did not decrease *Trim63* or *Fbxo32* gene expression, did not alter *Myod* expression, and did not increase Akt phosphorylation, suggesting that the preservation of myotube structure is unlikely to be explained by classical Akt–FOXO signaling pathways or by enhanced anabolic activity [40]. Instead, these findings suggest that preservation of myotube diameter reflects stabilization of myotube structure under conditions of metabolic stress. The mechanisms underlying this stabilization remain unclear. One possible explanation may involve a shift in cellular priorities away from energy‐demanding protein synthesis toward energy conservation and quality‐control processes. Such a shift could reflect metabolic adaptation processes that have previously been associated with AMPK activation under energetic stress, whereby cells reprioritize energy fluxes to sustain essential structural integrity rather than promoting hypertrophic growth [41, 42].

Western blot analysis showed that metformin increased AMPK phosphorylation at high concentrations, consistent with previous observations [43]. To further assess downstream AMPK signaling, we examined phosphorylation of acetyl‐CoA carboxylase (ACC), a canonical target of AMPK and a sensitive biochemical readout of its activity. Consistent with the AMPK findings, metformin promoted ACC phosphorylation, with the clearest effect observed at the high dose under simvastatin exposure and intermediate values at the low dose, supporting activation of AMPK‐associated signaling pathways.

### 4.4 Preservation of Mitochondrial Network Despite Reduced Respiratory Function

Our data show that metformin further suppressed both oxidative phosphorylation and glycolysis in simvastatin‐treated myotubes, as reflected by reductions in routine respiration, ATP‐linked respiration, and *Hk2* gene expression. Despite this overall decline in cellular energy production, mitochondrial network morphology was partially preserved, with increased branch length and network connectivity.

This dissociation between mitochondrial respiratory capacity and network architecture suggests that mitochondrial structural integrity can be maintained independently of short‐term respiratory output, a phenomenon that has been described in conditions of mitochondrial stress or AMPK activation [23]. Previous studies have shown that AMPK signaling can promote mitochondrial remodeling through coordinated regulation of mitochondrial fission, fusion, and selective removal of damaged mitochondrial components [24]. Such processes may lead to the formation of more interconnected mitochondrial networks while overall respiratory activity remains reduced. Maintenance of mitochondrial network integrity may confer advantages under energetic stress conditions. Interconnected mitochondrial networks are generally more resistant to cellular stress and can maintain mitochondrial membrane potential more effectively when energy supply is limited [44]. Moreover, mitochondrial fusion can facilitate complementation between partially damaged mitochondria, allowing more efficient utilization of remaining functional components [45, 46]. These observations suggest that mitochondrial structural resilience may represent an adaptive response to energetic stress. In this context, preservation of mitochondrial architecture may help sustain essential cellular functions during simvastatin‐induced metabolic stress and support recovery once energetic conditions improve.

### 4.5 Modulation of Cellular Stress Signaling by Metformin During Simvastatin Exposure

Our results demonstrate that metformin markedly reduced the expression of stress‐response genes (*Hri*, *Perk*, and *Atf4*) in simvastatin‐treated myotubes. These genes are core components of the ER stress–driven integrated stress response [47], which has been implicated in statin‐induced myotoxicity [48]. As the principal contributing factor, ATF4 suppresses global protein synthesis and activates stress‐adaptive programs. Excessive activation of the PERK–ATF4 axis promotes muscle atrophy by inhibiting translation and enhancing proteolysis [49, 50], whereas its inhibition can preserve protein synthesis and limit catabolic signaling [51, 52]. Our higher dose of metformin alone increased *Atf4* expression and reduced myotube size. In contrast, under simvastatin exposure, metformin suppressed *Atf4* gene expression, coinciding with partial preservation of myotube diameter.

Notably, the unfolded protein response activation is among the most energy‐demanding cellular responses [49, 53], and previous studies have shown that AMPK activation can reduce cellular energy load, modulating the unfolded protein response and attenuating ER stress [54]. Together, these findings suggest that the structural preservation of myotubes during simvastatin exposure may be associated with alterations in cellular stress signaling pathways, potentially linked to energy‐sensing mechanisms such as AMPK signaling.

### 4.6 Context‐Specific Effects on Proliferation and Differentiated Myotubes

Our finding that simvastatin markedly inhibited myoblast proliferation is in line with the observation that statins impair satellite cell activation and myoblast proliferation through induction of cell cycle arrest [55]. In contrast to its effects on differentiated myotubes, metformin did not restore simvastatin‐induced inhibition of proliferation. This observation aligns with the known anti‐proliferative effects of AMPK activation [56]. Satellite cell–derived myoblasts rely on proliferation and differentiation programs to sustain muscle regeneration, whereas post‐mitotic muscle fibers maintain mass primarily through signaling pathways such as Akt/mTOR, FOXO, and protein turnover [57]. Clinically, this implies that dual statin–metformin therapy may preferentially benefit mature myofibers, whereas support for regeneration after injury could require additional strategies tailored to proliferative compartments.

### 4.7 Practical Implications, Limitations, and Future Directions

Our findings suggest that metformin may partially attenuate certain structural and stress‐related responses to simvastatin in muscle cells. In particular, metformin helped preserve myotube diameter and aspects of myogenic integrity during simvastatin exposure. However, these protective effects were incomplete and occurred alongside further metabolic suppression, underscoring the complex interaction between these two commonly co‐prescribed drugs. From a clinical perspective, these observations may be relevant to statin‐associated muscle symptoms, especially given the high prevalence of coexisting dyslipidemia and diabetes in patients receiving combined statin and metformin therapy.

Several limitations should be acknowledged. First, although AMPK‐related signaling changes were observed, this study does not demonstrate that AMPK activation is responsible for the protective effects of metformin. Second, although supraphysiological concentrations of metformin are widely used in vitro to model intracellular drug accumulation and probe AMPK‐dependent mechanisms, the clinically relevant 50 μM dose showed limited efficacy in our system during the relatively short‐term exposure. Therefore, caution is warranted when extrapolating the protective effects observed at 1 000 μM to physiological conditions with much longer exposure times. Third, the C2C12 model lacks essential features of muscle tissue in vivo, including vascularization, neural input, immune interactions, and mechanical loading. Finally, the use of healthy myotubes may not fully reflect responses in hyperlipidaemia or aged muscle, and pathological culture conditions (e.g., lipid overload or aged myotubes) might yield different results.

Future studies should therefore investigate whether AMPK signaling contributes to these effects using direct genetic or pharmacological manipulation and validate these findings in more physiologically relevant models, including primary human myotubes and in vivo models of statin‐induced myopathy. Overall, the present findings highlight the complex metabolic interaction between statins and metformin in skeletal muscle cells and underscore the need for further mechanistic and translational studies.

## 5 Conclusion

In summary, simvastatin induced metabolic suppression, mitochondrial dysfunction, and activation of atrophy‐related pathways in differentiated muscle cells, resulting in reduced myotube diameter and impaired myogenic characteristics. Metformin partially attenuated several of these responses by preserving aspects of myotube structural integrity and reducing cellular stress signaling, despite further suppression of metabolic activity. These findings suggest that metformin may trigger adaptive metabolic responses that enhance cellular resilience during simvastatin‐induced metabolic stress. Further studies in more physiologically relevant systems are required to determine the implications of these interactions for skeletal muscle health in vivo.

## Author Contributions

**Chuqi He:** conceptualization; data curation; investigation; methodology; project administration; writing – original draft; writing – review and editing; visualization. **Mike Wesselink:** conceptualization; data curation; investigation; methodology; writing – review and editing. **Jelle Y. Huijts:** investigation; writing – review and editing. **Zhenjia Zhong:** data curation; investigation; methodology. **Moritz Eggelbusch:** methodology; writing – review and editing. **Richard T. Jaspers:** conceptualization; supervision; funding acquisition; writing – review and editing. **Rob C. I. Wüst:** conceptualization; supervision; funding acquisition; writing – review and editing.

## Funding

This research was funded by a grant from the China Scholarship Council (CSC grant no. 202207720075).

## Conflicts of Interest

The authors declare no conflicts of interest.

## Supporting information


**Figure S1:** Effects of simvastatin (Sim) and metformin (Met) on ACC signaling in C2C12 myotubes. Western blot analysis and quantification of (a) p‐ACC (Ser79)/pan‐actin ratio, (b) ACC/pan‐actin ratio. Pan‐actin was used as a loading control. Results are expressed as relative expression to control of independent experiments and represent the mean ± SEM (*N* = 6), ^#^ *p* < 0.05, ^##^ *p* < 0.001 presented the main effect between simvastatin and control.

## Data Availability

The datasets generated and analyzed during the current study are available from the corresponding author upon reasonable request.
