# Supplementary material for: Metformin Partially Attenuates Simvastatin‐Induced Myotoxic Responses in C2C12 Myotubes Through Metabolic Adaptation
Source: FASEB J. 2026 Jun 26;40(13):e72075. doi: 10.1096/fj.202600077RRR (PMC13308772; doi:10.1096/fj.202600077RRR)
Supplement: Supplementary file 1 — Figure S1: Effects of simvastatin (Sim) and metformin (Met) on ACC signaling in C2C12 myotubes. Western blot analysis and quantification of (a) p‐ACC (Ser79)/pan‐actin ratio, (b) ACC/pan‐actin ratio. Pan‐actin was used as a loading control. Results are expressed as relative expression to control of independent experiments and represent the mean ± SEM (N = 6), # p < 0.05, ## p < 0.001 presented the main effect between simvastatin and control. [file FSB2-40-e72075-s001.docx]

**Metformin partially attenuates simvastatin-induced myotoxic responses in C2C12 myotubes through metabolic adaptation**

**Chuqi He^1^, Mike Wesselink^1^, Jelle Y Huijts^1^, Zhenjia Zhong^1^, Moritz Eggelbusch^1,2^, Richard T Jaspers^1^, Rob CI Wüst^1*^**

1 Department of Human Movement Sciences, Faculty of Behavioural and Movement Sciences, Amsterdam Movement Sciences, Vrije Universiteit Amsterdam, Amsterdam, The Netherlands

2 Professorship of Exercise Biology, Department Health and Sport Sciences, TUM School of Medicine and Health, Technical University of Munich, Munich, Germany.

*: Address for correspondence: Rob CI Wüst PhD, Department of Human Movement Sciences, Faculty of Behavioural and Movement Sciences, Vrije Universiteit Amsterdam, Van der Boechorststraat 7, 1081 BT Amsterdam, The Netherlands. E-mail address: [r.wust@vu.nl](mailto:r.wust@vu.nl). Phone number: +31621183368

**
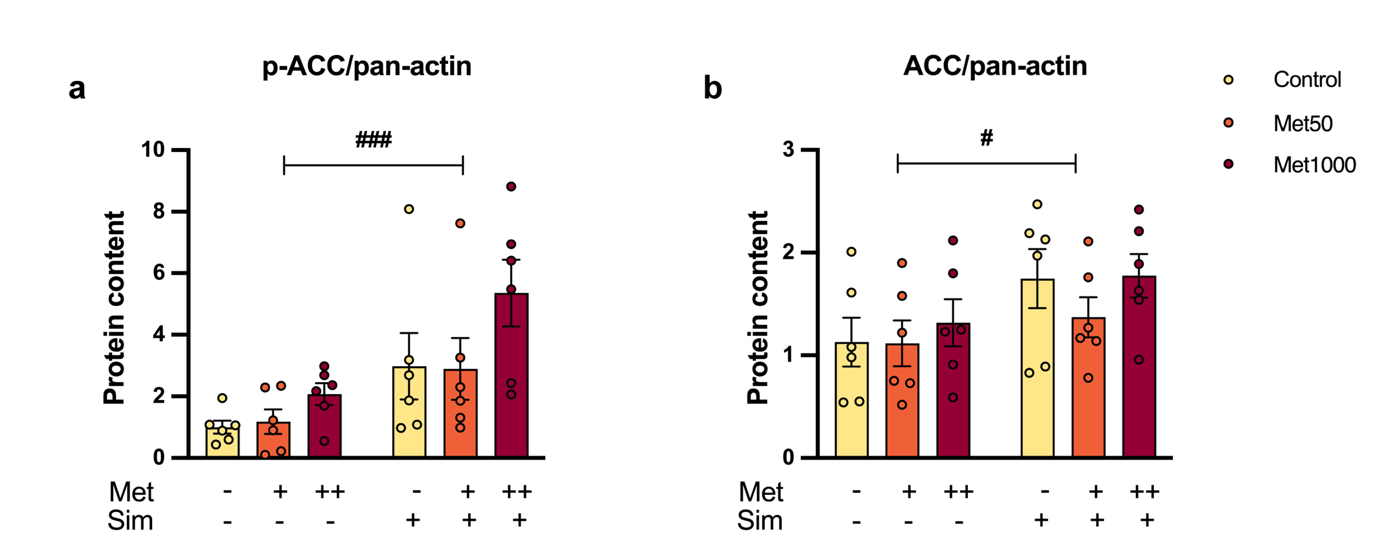
**

**Supplement Fig.S1 Effects of simvastatin (Sim) and metformin (Met) on ACC signaling in C2C12 myotubes.** Western blot analysis and quantification of **(a)** p-ACC (Ser79)/pan-actin ratio, **(b)** ACC /pan-actin ratio. Pan-actin was used as a loading control. Results are expressed as relative expression to control of independent experiments and represent the mean ± SEM (N=6), ^#^P < 0.05, ^###^P < 0.001 presented the main effect between simvastatin and control.
